# Clinical trial for the control of water intake of patients undergoing hemodialysis treatment*

**DOI:** 10.1590/1518-8345.2694.3091

**Published:** 2018-11-29

**Authors:** Graziella Allana Serra Alves de Oliveira Oller, Marília Pilotto de Oliveira, Cláudia Bernardi Cesarino, Carla Regina de Souza Teixeira, José Abrão Cardeal da Costa, Luciana Kusumota

**Affiliations:** 1 Universidade Paulista, São José do Rio Preto, SP, Brazil.; 2 Universidade de São Paulo, Escola de Enfermagem de Ribeirão Preto, PAHO/WHO Collaborating Centre for Nursing Research Development, Ribeirão Preto, SP, Brazil.; 3 Faculdade de Medicina de São José do Rio Preto, Departamento de Enfermagem Geral, São José do Rio Preto, SP, Brazil.; 4 Universidade de São Paulo, Faculdade de Medicina de Ribeirão Preto, Ribeirão Preto, SP, Brazil.

**Keywords:** Renal Insufficiency, Chronic, Renal Dialysis, Clinical Trial, Health Education, Weight Gain, Nursing

## Abstract

**Objective:**

to analyze the impact of an educational and motivational intervention for patients with a chronic kidney disease, undergoing hemodialysis treatment, on the control of fluid intake during interdialytic periods.

**Method:**

a quasi-experimental, non-randomized clinical trial with patients from a Nephrological Unit of the State of São Paulo. Participants were included in two groups: Control Group with 106 patients and Intervention Group with 86 patients, totaling 192 participants. The used intervention was an educational and motivational video to control liquid intake, based on the Bandura’s Theory. The measure of control of water intake was the percentage of lost weight, also considered the variable outcome of the research. For the data analysis, descriptive analyses and regression analysis of the Inflated Beta Model were used.

**Results:**

patients who participated in the intervention had a decrease in the pattern of weight gain in interdialytic periods, with a 3.54 times more chance of reaching the goal of 100% of weight loss when compared to participants from the control group.

**Conclusion:**

the educational and motivational intervention was effective in reducing the percentage of weight loss in patients undergoing hemodialysis. Brazilian Clinical Trials Registry (ReBEC) under the opinion RBR-4XYTP6.

## Introduction

Many of the problems experienced by patients with Chronic Kidney Disease (CKD) undergoing hemodialysis treatment are related to a low adherence to the proposed treatment. Fluid overload is common for these patients, and their excess is linked to an increase in the morbidity of this population^1^ ^-^ ^2^.

Although it is regulated by physiological mechanisms, the behavior of fluid intake is also influenced by the person’s habits, customs, and social rituals, as well as other factors that trigger water intake, such as a deficit in extracellular volume and blood pressure, and lack of moisture of the oral mucosa and esophagus^3^. Thirst plays a role in the maintenance of fluid homeostasis, which implies a network of complex neural and hormonal processes in response to an imbalance in the body’s water and sodium ratio^4^ ^-^ ^5^.

Many patients with CKD undergoing hemodialysis treatment intake more fluids than recommended, a practice that is common among these persons^6^. The management of liquid intake is a challenge for most patients since, in addition to the liquids, many foods have a high water content, such as fruits, jellies, and soups^7^ ^-^ ^9^. Approximately 95% of the patients with CKD undergoing hemodialysis do not adhere to the prescribed treatment for water restriction, which can lead to many complications^10^.

Patient education is one of the most useful, effective, and accessible health care tools^11^. Health behavior change is described as the result of reciprocal relationships between the environment, personal factors, and the attributes of one’s behavior^12^.

The theory underlying the elaboration of educational material and its implementation in this study was the Cognitive-Social Theory, whose basic principle is the perspective of human agency for self-development, adaptation, and change. Human thought and action are considered as products of a dynamic interrelationship between personal, behavioral, and environmental influences, which enables targeted therapeutic interventions^13^.

Several are meanings passing through the imaginary of patients with CKD, from the impact of diagnosis, associated with the recognition of disease severity and treatment, to its consequences, such as the medicinal effects and limits on diet and water habits^14^. Changes in living habits because of illness generate difficulties associated with the absence of experiences that provide pleasure, physical incapacity to perform daily activities, travel to dialysis centers, and changes in working and financial conditions. They also refer to concerns about venous access, water control, and dietary restrictions. In general, these situations cause doubts, insecurities, fears, anguishes, and sufferings regarding healing and the possibility of living^15^.

Thus, this study aimed to analyze the impact of an educational and motivational intervention for patients with chronic kidney disease undergoing hemodialysis treatment on the control of fluid intake during interdialytic periods.

## Method

A quasi-experimental, non-randomized clinical trial was conducted with patients undergoing hemodialysis treatment at a Nephrology Unit in the State of São Paulo, Brazil. The unit serves 346 patients undergoing hemodialysis treatment, continuous ambulatory peritoneal dialysis (CAPD) and automated dialysis (APD). In the hemodialysis service, patient care is given in shifts, with sessions of four hours each, on Mondays, Wednesdays, and Fridays or on Tuesdays, Thursdays, and Saturdays.

**Figure 1:**
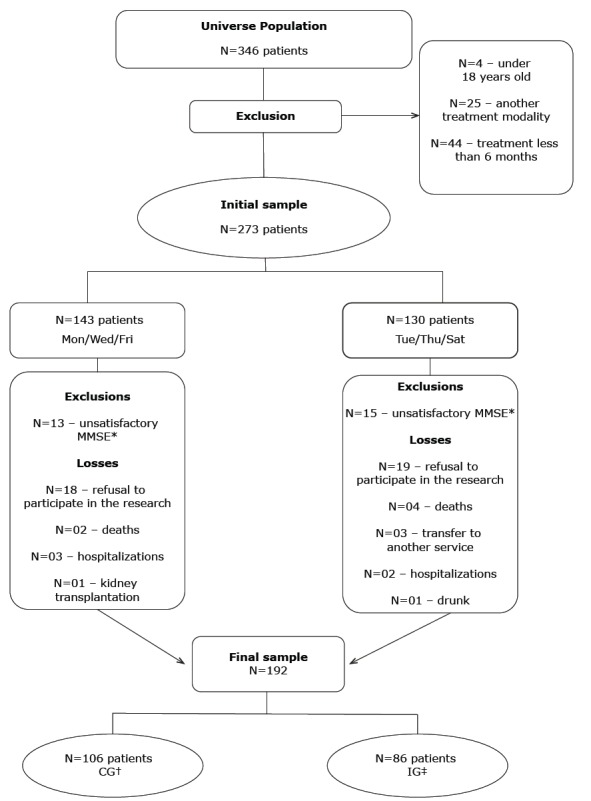
Design of the study participants

Note: *MMSE - Mini-Mental State Examination; †CG - Control Group; ‡ IG - Intervention Group

A total of 273 patients were eligible for the study, being divided into two groups: Intervention Group (IG), with 86 patients undergoing hemodialysis on Tuesday, Thursday, and Saturday; and a Control Group (CG), with 106 patients undergoing hemodialysis on Monday, Wednesday, and Friday. This measure was adopted in order to minimize the bias of confusion regarding persons who could meet and exchange information about each other on the intervention. The study design and data collection steps are described in Figure 1.

The following instruments were used for data collection: 1) Instrument for the characterization of socio-demographic, economic, and clinical data; 2) Instrument of General and Perceived Self-efficacy, which is a scale translated and adapted to Brazil in 2004 with the objective of predicting the person’s ability to overcome difficulties experienced daily, as well as his/her adaptation after the experience of adverse life events^16^; 3) Folkman and Lazarus Coping Strategies Inventory (FLCSI), translated and validated for Brazilian Portuguese in 1995, covering all methodological steps for translation, validation, and cultural adaptation of evaluation instruments. The results of tests indicated that the instrument is accurate and valid for the study of coping strategies and that its application allows identifying ways of dealing with stress^17^; 4) Resilience Scale, which was translated, adapted, and validated for Brazil in 2005 and has the objective of measuring the levels of positive psychosocial adaptation in face of important life events^18^; 5) Hospital Anxiety and Depression Scale (HAD), translated and validated for Brazil in 1995, this scale aims to detect mild degrees of affective disorders in non-psychiatric settings, in several contexts^19^. These instruments were determined as the independent variables of the study.

The ideal percentage of weight loss was considered as a variable outcome of the study and was determined by the pre- and post-dialysis weight measurements, as well as the dry weight of each participant, during the study period. Interdialytic weights data collection and follow-up of patients lasted 16 sessions, with pre- and post-dialysis data recorded by the researcher during the data collection.

A digital media was planned and elaborated in a video format, which was used as educational and motivational strategy, composing the intervention of the study. For the construction of the educational material, a search was made in the scientific literature and in protocols of dialysis service on the topics to be addressed. Thus, an educational video, called *“Motivational Intervention - How my kidneys work: treatment and weight gain”*, was developed based on the Cognitive-Social Theory^13^. The following topics were addressed: location and functions of the kidneys; CKD and impairment of kidneys; hemodialysis; guidance on fluid consumption and dialysis complications; measures and care to maintain the hydroelectrolytic balance.

Data collection took place from January to April 2017. The study was conducted over a five-week follow-up period with each patient. The intervention was performed during two meetings, with two more moments for reinforcement with IG patients during hemodialysis, with the stable procedure.

At the first meeting (T_0_), the research participants were invited to participate in the research after being informed about it, its relevance, and duration of data collection. At this step, sociodemographic and clinical information were collected, as well as measured the variables of self-efficacy, coping, resilience, and symptoms of anxiety and depression. All CG and IG patients participated in this step.

At the second meeting (T_1_), one week after T_0_, the first intervention session was conducted with only the participants of IG. The aim of this step was to provide information on hemodialysis treatment and its complications, demonstrating the effects of excessive water intake in patients with CKD. The educational video was presented in digital format on a tablet. One week later, the first reinforcement (T_2_) occurred, in which a face-to-face approach was taken by the researcher to reinforce the intervention and follow up of the guidelines through feedback. At this step, a dialogued meeting was held between the researcher and the patient in order to verify what the patient in the first intervention has learnt. The patient was encouraged to verbalize the content of the intervention, which led to the realization of complementary guidelines related to possible doubts about the educational and motivational video. This step was performed only with IG participants.

The second session of the intervention occurred one week after the first reinforcement (T_3_) to intensify the information on the hemodialysis treatment and its complications and discuss strategies for the reduction of water intake only with IG participants. The researcher gave verbal guidance and presented the educational video again. Strategies for the reduction of water intake were discussed. Three weeks after the first intervention, a new reinforcement (T_4_) was performed in a dialogued meeting with the patient to discuss all the issues addressed since the beginning of the study, reinforce, and maintain what was learned.

At the last meeting (T_5_), the psychosocial variables of interest (self-efficacy, coping, resilience, and symptoms of anxiety and depression) were measured again by means of an interview with CG and IG patients. The CG patients were submitted to the same measures of the variables of interest in the first and sixth steps and weighed at all intervals as in IG. After completing the data collection, the patients had access to the educational and motivational video.

**Figure 2:**
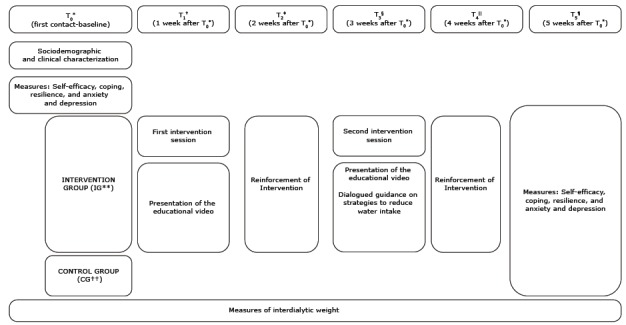
Steps of the study according to the five-week follow-up for data collection and implementation of the intervention

Note: *T_0_-baseline; †T_1_- one week after T_0_*; ‡T_2_-two weeks after T_0_*; §T_3_-three weeks after T_0_*; ||T_4_-four weeks after T_0_*; ¶T_5_-five weeks after T_0_*; **IG-Intervention Group; ††CG-Control Group

The data collection of interdialytic weights and patient follow-up lasted 16 sessions (P1 to P16). Interdialytic weight gain was calculated as the difference between the current pre-dialysis weight and the previous post-dialysis weight, i.e. the weight in which the patient started the session to be performed minus the weight at the end of the previous session. Afterward, the measurements were transformed into the “ideal percentage of weight loss”, being considered as the variable outcome. Thus, a better visualization on how close to the ideal weight loss the patient reached was obtained. The ideal percentage of weight loss was calculated by the ratio between the actual difference (pre-dialysis weight − post-dialysis weight) and the ideal weight difference (pre-dialysis weight − dry weight). The more the patient approaches 100%, the closer he/she is to the ideal weight loss. The measures P4 and P10 corresponded to the stages of implementation of the intervention T1 and T3 of the study. Thus, a better visualization on how close to the ideal weight loss the patient reached was obtained. The steps of data collection are shown in Figure 2.

The data analysis was performed using the statistical software SAS 9.0 and the program R version 3.4.1. A descriptive analysis was performed for the categorical variables by means of simple frequency, and the numerical variables were analyzed according to the measures of central tendency and dispersion. For the analysis of the variable outcome, a regression analysis of the Inflated Beta Model was performed^20^. The Shapiro-Wilk test was applied to the residuals of the model to verify the hypothesis of their normality.

Confidence intervals with 95% probability were used and the significance level adopted was 5%.

This research was approved by the Research Ethics Committee of the Ribeirão Preto College of Nursing - EERP/USP, according to Resolution 466/2012 of the National Health Council for research involving human beings under the CAAE: 54339616.6.0000.5393 and Opinion: 1.689.258. Subsequently, the Research Ethics Committee of FAMERP approved the research, where data were collected under the CAAE: 54339616.6.3001.5415 and Opinion: 1.887.840. The research was included in the Brazilian Clinical Trials Registry (ReBEC) under the opinion RBR-4XYTP6.

## Results

A total of 192 patients participated in the study, 106 in GC and 86 in IG. A predominance of male participants was observed in both groups (CG = 57.6%; IG = 66.3%). Age ranged from 18 to 90 years, with an average of 57.6 years in CG and 58.9 years in IG. In relation to the marital status, the participants were predominantly married or living in a consensual union (CG = 68.9%; IG = 76.7%). Regarding schooling, a predominance of patients who could read and write was observed (CG = 91.5%; IG = 87.2%), with an average of 7.6 years of study in CG and 6.8 years of study in IG. Few patients reported having their own work as income (CG = 2.8%; IG = 11.6%). The majority of the participants supported themselves with retirement (CG = 50.9%; IG = 64%).

The self-reported diseases were arterial hypertension (CG = 81.1%; IG = 68.6%), visual deficit (CG = 70.8%; IG = 57%), diabetes mellitus (CG = 50.9%; IG = 43.0%), among others. The average number of self-reported diseases per patient was 3.4 in CG and 2.9 in IG. Among the physical complications related to CKD and hemodialysis treatment, the most cited by patients were cramps (CG = 83%; IG = 80.2%), anemia (CG = 80.2%; IG = 75.6%), and arterial hypotension during the dialysis session (CG = 79.3%; IG = 73.3%). The average number of complications per patient in CG was 6.2 and 7.4 in IG.

Regarding the variable outcome, a tendency to increase the ideal percentage of weight loss during the sessions was observed for all patients, which approached the goal of 100% lost weight. The variability was lower in IG when compared to CG whereas the ideal percentage of weight loss in patients who reached the goal in IG was higher when compared to CG, except for the initial time (Table 1).

**Table 1:** − Description of the 16 measures of the variable weight of the 192 patients undergoing hemodialysis treatment according to CC* and IG^†^. São José do Rio Preto, SP, Brazil, 2017

Time	CG* (n=106)						IG^†^ (n=86)					
	Minimum	Median	Mean	Maximum	SD^‡^	Ideal % of weight loss	Minimum	Median	Mean	Maximum	SD^‡^	Ideal % of weight loss
P1	29.55	100.00	94.72	100.00	10.52	0.5849	70.69	96.92	95.18	100.00	6.26	0.4884
P2	50.00	100.00	95.38	100.00	8.46	0.5755	84.21	100.00	96.76	100.00	4.60	0.5814
P3	50.41	100.00	95.73	100.00	8.59	0.6321	83.05	100.00	97.46	100.00	4.42	0.6744
P4	48.76	100.00	95.62	100.00	8.68	0.6321	81.40	100.00	97.08	100.00	4.54	0.6512
P5	49.59	100.00	96.03	100.00	8.38	0.6604	66.67	100.00	98.97	100.00	4.32	0.9186
P6	48.00	100.00	95.86	100.00	8.65	0.6698	71.43	100.00	99.21	100.00	3.35	0.8721
P7	48.03	100.00	95.95	100.00	8.51	0.6321	70.59	100.00	98.90	100.00	3.65	0.8372
P8	49.59	100.00	95.19	100.00	9.31	0.6321	54.84	100.00	98.66	100.00	5.72	0.8953
P9	47.93	100.00	95.53	100.00	9.42	0.6698	81.25	100.00	99.28	100.00	2.49	0.8721
P10	49.59	100.00	95.25	100.00	9.17	0.6226	54.55	100.00	98.24	100.00	5.95	0.8140
P11	47.58	100.00	95.19	100.00	9.12	0.5849	41.18	100.00	98.40	100.00	7.16	0.8837
P12	50.41	100.00	95.39	100.00	9.08	0.6415	71.43	100.00	99.45	100.00	3.18	0.9186
P13	51.67	100.00	95.27	100.00	9.19	0.6132	78.12	100.00	99.13	100.00	3.31	0.8953
P14	47.50	100.00	95.54	100.00	9.11	0.6226	82.35	100.00	99.38	100.00	2.61	0.9302
P15	47.93	100.00	95.41	100.00	9.34	0.6226	75.00	100.00	98.95	100.00	3.74	0.8837
P16	51.61	100.00	95.76	100.00	8.69	0.6415	87.10	100.00	99.54	100.00	2.08	0.9302

Note: *CG - Control Group; †IG - Intervention Group; ‡SD - Standard Deviation

**Figure 3:**
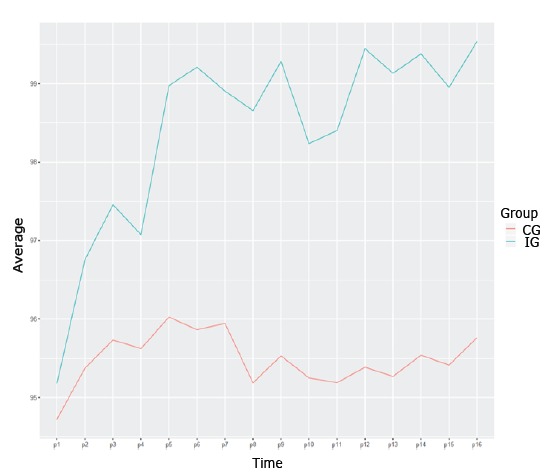
Distribution of the ideal percentage of weight loss by groups (CG* and IG^†^) over time

Note: *CG - Control Group; †IG -Intervention Group

Figure 3 shows the progression of the ideal percentage of weight loss in the study period. The increase in the ideal percentage of weight loss stands out in GI, especially from the fifth measurement (P5), which corresponded to the first weight measure after the implementation of the first educational and motivational intervention. In view of these results, it is possible to affirm that the educational and motivational intervention had a positive impact on the control of fluid intake, as measured by the ideal percentage of weight loss of patients undergoing hemodialysis treatment.

A regression analysis of the Inflated Beta Model was performed between the outcome and independent variables. In Model 1 of the regression, resulting from the modeling for the parameter μ, the independent variables (groups CG and IG, years of study, time of diagnosis of CKD, time of hemodialysis, number of comorbidities, self-efficacy, the factors confrontation, self-control, social support, escape-avoidance, and positive reevaluation of FLCSI, resilience, depression symptoms, and ideal percentage of weight loss in the 16 times) were related to the outcome, but the model was rejected since the adjustment was not satisfactory for the Shapiro-Wilk test, which indicated rejection of the normality hypothesis for the residuals (W = 0.9983, p-value = 0.0020).

In Model 2, the independent variables were inserted for dispersion, maintaining all variables of the first step. This modeling for the parameter σ was satisfactory for the Shapiro-Wilk normality test and indicated non-rejection of the normality hypothesis for the residuals (W = 0.9999, p-value = 0.3446).

However, we proposed the Model 3, choosing to add the modeling to the parameter τ, i.e. the probability of reaching the goal (losing 100% of the stipulated weight). For this model, the significant variables of μ and σ obtained in Model 2 were maintained. The significant variables for τ were Group (CG/IG) and the ideal percentage of weight loss in the 16 times, as well as the scores of the scales of resilience, anxiety symptoms, and the factors confrontation, self-control, social support, acceptance of responsibility, and positive reevaluation of FLCSI. All the independent variables inserted in Model 3 showed a statistical significance, except for the variable “weight 2”.

For the variable Group (CG/IG), IG participants were 3.54 times more likely to reach the goal when compared to CG. At time 3 of weight measurement, participants were 1.67 times more likely to achieve the goal when compared to time 1 (baseline). The interpretation is analogous to the other times (Table 2).

**Table 2:** Regression Analysis of the Inflated Beta Model for the parameter scale, maintaining the significant variables for the mean and dispersion of the 192 patients undergoing hemodialysis treatment. São José do Rio Preto, SP, Brazil, 2017

Scale Parameters (τ*)	Estimate	Standard error	p-value^‡^	τ*	95%^§^ CI τ*	
(Intercept)	−1.6424	0.4419	0.0002	0.1935	0.0814	0.4601
Weight 2	0.1666	0.2183	0.4454	1.1813	0.7701	1.8119
Weight 3	0.5125	0.2220	0.0210	1.6695	1.0805	2.5796
Weight 4	0.4616	0.2213	0.0371	1.5866	1.0283	2.4481
Weight 5	1.1975	0.2379	0.0000	3.3118	2.0776	5.2791
Weight 6	1.1013	0.2349	0.0000	3.0081	1.8983	4.7669
Weight 7	0.8912	0.2292	0.0001	2.4380	1.5556	3.8209
Weight 8	1.0394	0.2331	0.0000	2.8275	1.7906	4.4650
Weight 9	1.1013	0.2349	0.0000	3.0081	1.8983	4.7669
Weight 10	0.8061	0.2273	0.0004	2.2391	1.4341	3.4958
Weight 11	0.8625	0.2286	0.0002	2.3691	1.5136	3.7080
Weight 12	1.1329	0.2358	0.0000	3.1047	1.9555	4.9291
Weight 13	0.9791	0.2315	0.0000	2.6620	1.6912	4.1900
Weight 14	1.1013	0.2349	0.0000	3.0081	1.8983	4.7669
Weight 15	0.9791	0.2315	0.0000	2.6620	1.6912	4.1900
Weight 16	1.1650	0.2368	0.0000	3.2058	2.0153	5.0997
Group IG^||^	1.2642	0.1097	0.0000	3.5404	2.8555	4.3895
Self-efficacy	−0.0369	0.0078	0.0000	0.9638	0.9492	0.9787
Confrontation Factor (FLCSI^¶^)	0.7270	0.0830	0.0000	2.0689	1.7582	2.4345
Escape-Avoidance Factor (FLCSI^¶^)	−0.1924	0.0609	0.0016	0.8250	0.7321	0.9296
Problem solving factor (FLCSI^¶^)	0.2868	0.0855	0.0008	1.3322	1.1265	1.5753
Positive Reevaluation Factor (FLCSI^¶^)	−0.3789	0.1078	0.0004	0.6846	0.5542	0.8457
Resilience	0.0200	0.0032	0.0000	1.0203	1.0138	1.0267
Symptoms of Anxiety	−0.0399	0.0168	0.0179	0.9609	0.9297	0.9931

Note: *τ - Scale parameters, maintaining the significance obtained in mean and scale; ‡p-value - Regression Analysis of the Inflated Beta Model; §95% CI - 95% confidence interval; ||GI - Intervention Group; ¶FLCSI - Folkman and Lazarus Coping Strategies Inventory

An increase was observed in the ideal percentage of weight loss of IG patients when compared to CG. Patients with CKD undergoing hemodialysis treatment who participated in the educational and motivational intervention on fluid intake decreased the pattern of weight gain in interdialytic periods.

## Discussion

Education affects the way people behave when facing adversity. Educational practices are effective when they influence the beliefs people have in their own capacities^21^. In this study, the positive impact of an educative and motivational intervention elaborated and implemented for the control of fluid intake for patients with CKD undergoing hemodialysis treatment was observed.

The adequate choice of the tool for the development of this educational intervention, coupled with the adoption of a theoretical reference, was essential for conducting the intervention. In this sense, the theoretical reference should allow the favoring of the teaching-learning process.

The intervention used in this study was elaborated according to Bandura’s Social Cognitive Theory^13^. Patients from IG participated in an educational intervention with the demonstration of a video during the hemodialysis session and dialogues were held to reinforce guidelines on CKD and water intake control.

A study conducted in 2015 aiming at identifying the factors that contribute to the adherence to the diet of patients with CKD undergoing hemodialysis found that 25% of them did not have adherence to the prescribed treatment and that, in 86% of cases, it influences the morbidity and mortality of this population. Age, dialysis time, motivation, and distorted perception of adherence to treatment were factors raised as intrinsic barriers to the adherence to diet and water prescription, while self-efficacy, disease perception, and disease control perception were facilitators of the treatment^22^.

There are still few studies with a theoretical basis in Bandura with regard to patients with CKD. However, international researches have been increasingly interested in the development and use of interventions to promote and support patients in relation to health care^23^ ^-^ ^24^.

In health settings, patients report thirst as one of the most prevalent and uncomfortable symptoms, which can overcome all other sensations^25^. When sodium balance is well controlled, thirst mechanism adequately regulates water balance^26^.

Salt intake is a relevant factor in interdialytic weight gain. Patients undergoing hemodialysis treatment receive more fluid in response to the sensation of osmotic thirst, which is usually caused by sodium ingestion^27^ ^-^ ^28^. Immediately after the hemodialysis session, patients may also suffer from volumetric thirst caused by hypovolemia related to the ultrafiltration process^27^.

However, during hemodialysis, there may also be a diffuse sodium transfer to the patient. Some authors attribute an increased thirst in patients with CKD undergoing hemodialysis treatment to sodium dialysate prescription. They state that the diffuse sodium transfer to the patient during hemodialysis contributes to the incomplete removal of sodium and that this problem could be minimized with an individualized prescription of sodium dialysate^29^ ^-^ ^30^.

A study carried out with patients undergoing hemodialysis showed that those who underwent hemodialysis sessions with a lower dialytic sodium concentration had a lower interdialytic weight gain and lower blood pressure values. The authors state that changes in sodium dialysate concentration may contribute to a reduction in interdialytic weight gain^31^.

Dietary and dialytic sodium restriction may possibly contribute to decreasing volume overload in patients undergoing hemodialysis^32^ ^-^ ^33^. Thus, an adequate sodium balance should compose a goal associated with the control of water intake for these patients.

In addition, a randomized controlled clinical trial conducted in the State of Paraíba, Brazil, with 60 patients with CKD undergoing hemodialysis should be mentioned. The authors evaluated the effect of a musical intervention on anxiety and vital parameters in this population and found a statistically significant reduction of the anxiety score after musical hearing (p=0.03), as well as systolic blood pressure (p<0.002), diastolic blood pressure (p<0.002), heart rate (p<0.01), and respiratory rate (p<0.006). Thus, they demonstrated the musical intervention as a therapeutic resource likely to be used, evidencing the importance of the use of complementary practices by nurses in their daily life^34^.

The scarcity of intervention studies with patients with CKD, in particular researches that seeks to investigate water intake control, is evident. Therefore, the educational and motivational intervention used in this study was effective for the control of fluid intake in interdialytic periods of patients with CKD undergoing hemodialysis treatment. In addition, an increase in the ideal percentage of weight loss was observed among the patients who participated in the educational and motivational intervention, being possible to affirm that the intervention had a positive impact on the fluid intake control.

As study limitations, there is a non-randomization since patients undergoing hemodialysis perform the treatment on fixed days and times and interact during the sessions, thus establishing contact between IG and CG. If randomized, they could result in biases in outcome evaluations. The financial investment for the production of the educational and motivational video may be another aspect to be considered as limiting to the reproduction of this intervention in some health services. The segment time of the study was considered satisfactory.

## Conclusion

There was a positive impact of the educational and motivational intervention in the control of fluid intake, evaluated through interdialytic weight gain measures of patients with CKD undergoing hemodialysis treatment. An increase in the ideal percentage of weight loss was observed during the sessions, i.e. after the participation in the intervention, the patients had an ideal percentage of weight loss that was closer to that recommended (100%). Patients who participated in the intervention had a reduction in the pattern of weight gain in interdialytic periods, with a 3.54 times more chance of reaching the goal of 100% of weight loss when compared to participants in the control group. There is an increase in the ideal percentage of weight loss of patients of IG when compared to CG.

The educational and motivational intervention was considered positive and adequate to be used in the hemodialysis services since it contributes to the achievement of ideal goals of maintaining the interdialytic weight. Further studies of intervention and use of communication technologies are commendable, and nursing planning can be included in them to subsidize improvements in care delivery.
